# Second-generation proteasome inhibitor carfilzomib sensitizes neuroblastoma cells to doxorubicin-induced apoptosis

**DOI:** 10.18632/oncotarget.12427

**Published:** 2016-10-04

**Authors:** Shan Guan, Yanling Zhao, Jiaxiong Lu, Yang Yu, Wenjing Sun, Xinfang Mao, Zhenghu Chen, Xin Xu, Jessie Pan, Surong Sun, Jianhua Yang

**Affiliations:** ^1^ Xinjiang Key Laboratory of Biological Resources and Genetic Engineering, College of Life Science and Technology, Xinjiang University, Urumqi 830046, Xinjiang, China; ^2^ Texas Children's Cancer Center, Department of Pediatrics, Dan L. Duncan Cancer Center, Baylor College of Medicine, Houston, TX 77030, USA; ^3^ Department of Ophthalmology, Shanghai Tenth People's Hospital, Tongji University School of Medicine, Shanghai 200072, China; ^4^ Labratory of Medical Genetics, Harbin Medical University, Harbin, Heilongjiang 150081, China

**Keywords:** neuroblastoma, proteasome inhibitor, carfilzomib, chemotherapy, doxorubicin

## Abstract

Neuroblastoma (NB), which accounts for about 15% of cancer-related mortality in children, is the most common extracranial malignant neoplasm in children. Elevated level of proteasome activity promotes cancer development and the inhibition of proteasome activity is a promising strategy for cancer treatment. Therefore, targeting proteasome by small molecule inhibitors may be a viable option for NB therapy. Here in this study, we show that a novel proteasome inhibitor Carfilzomib (CFZ) exerts anti-tumor effect on NB. CFZ caused decreased cell viability and attenuated colony formation ability of a subset of NB cell lines. CFZ induced cell apoptosis in NB cells. Moreover, CFZ enhanced the cytotoxic effect of doxorubicin (Dox) on NB cells and Dox-induced p38 and JNK phosphorylation. In addition, CFZ inhibited Dox-induced NF-κB activation by stabilizing the protein level of IκBα. Furthermore, CFZ induced apoptosis and augmented Dox-induced apoptosis in NB tumor cells in orthotopic xenograft mouse models. In summary, our study suggests that proteasome is a therapeutic target in NB and proteasome inhibition by CFZ is a potential therapeutic strategy for treating NB patients.

## INTRODUCTION

Ranking as the most common childhood extracranial neoplasm, neuroblastoma (NB) accounts for about 15% of cancer-related deaths in children [1–3]. With the collaborations of cooperative group trials in US and around the world, low- and intermediate-risk NB have demonstrated good outcomes. Nevertheless, overall survival rate for high-risk NB remains poor. Thus, novel therapies for NB, especially high-risk NB, are urgently needed.

Proteasome, which plays a crucial role in cellular homeostasis, participates in intracellular protein degradation via ubiquitin/proteasome pathway [4–9]. The inhibition of proteasome functions, especially selective inhibition of proteasome's chymotrypsin-like subunits, would promote cell cycle arrest, apoptosis and cell death with minimal side-effects on total cellular protein turnover, which in turn helps to substantiate proteasome as an optimal chemotherapeutic target [10–15].

The transcription/nuclear factor kappa B (NF-κB), is involved in the regulation of immune/inflammatory responses, proliferation, and tumorigenesis [16–18]. Elevated NF-κB activation in cancer cells promotes tumor survival and progression [19–21]. In NB, the up-regulation of NF-κB has been shown to accelerate tumor growth and promote cancer cell survival [1, 19, 22, 23]. The blockade of NF-κB activation in cancer cells has been suggested to be a potential therapeutic strategy [24, 25]. The nuclear factor of kappa light polypeptide gene enhancer in B-cells inhibitor, alpha (IκBα) is one member of a family of cellular proteins that function to inhibit the NF-κB transcription factor. Since the ubiquitination and proteasomal degradation of IκBα is required for NF-κB activation, and proteasome inhibition augments chemotherapeutic response by inhibiting IκBα degradation and subsequently inhibiting NF-κB activation [10–15].

Two FDA (US Food and Drug Administration)-approved proteasome inhibitors, Bortezomib (for treating multiple myeloma) and Ixazomib (for treating relapsed/refractory myeloma, in combination with lenalidomide and dexamethasone), demonstrate significant anti-tumor effects majorly via blocking the activation of NF-κB pathway [19, 26–29], and are regarded as promising anti-NB regimen [26]. However, Bortezomib failed to produce objective responses *in vivo* on chronic lymphocytic leukemia and resulted in severe cytotoxicities to normal tissue [30]. And despite proteasome inhibitors being highly active, resistance is commonly observed [31, 32]. Therefore, novel therapeutic agents with improved efficacy need to be developed.

Carfilzomib (CFZ) is a novel proteasome inhibitor that has already been approved by the FDA for treating the relapsed and refractory multiple myeloma in July of 2012 [33–36]. Prior studies demonstrated that CFZ irreversibly inhibits 26S proteasome activity and efficiently stabilize IκBα by inhibiting its degradation, subsequently inhibiting NF-κB activation and inducing apoptosis pathway [37–39]. In addition, CFZ activated the members of MAPK family, including the stress-activated kinases p38, JNK, and ERK1/2 in leukemia/lymphoma, lung cancer [40], etc. Herein, we evaluate the cytotoxic effects of CFZ on NB cells. Our results demonstrate that CFZ induced apoptosis and enhanced doxorubicin (Dox)-induced apoptosis through inhibiting the NF-κB activation and activating p38 and JNK pathway in NB. Our studies suggest that novel proteasome inhibitor CFZ might be a potential therapeutic agent for NB patients.

## RESULTS

### Proteasome inhibitor CFZ exhibits cytotoxic effect on NB cells

**Figure 1:**
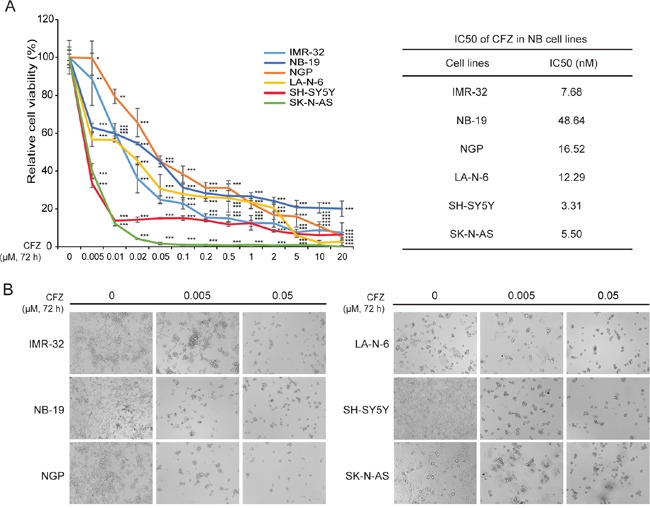
CFZ shows cytotoxic effect on a panel of six NB cells **A.** Six NB cell lines were treated with increasing concentrations (0 μM, 0.005 μM, 0.01 μM, 0.02 μM, 0.05 μM, 0.1 μM, 0.2 μM, 0.5 μM, 1 μM, 2 μM, 5 μM, 10 μM, 20 μM) of CFZ for 72 h. Cell viability was assessed by CCK-8 assay. *P*<0.05 (*), *P*<0.01 (**), or *P*<0.001 (***) (Student's *t*-test, two-tailed) as indicated. The IC50 values of CFZ in each cell line listed were calculated in Graphpad Prism 5 and based on the data collected in the cell viability assay. **B.** Morphological changes of the six different NB cell lines treated with increasing concentrations (0 μM, 0.005 μM, 0.05 μM) of CFZ for 72 h were shown.

To determine the potential cytotoxic effect of CFZ on NB cells, the CCK-8 assay was measured on six NB cell lines, including three N-myc amplified cell lines (IMR-32, NB-19, NGP) and three N-myc non-amplified cell lines (LA-N-6, SH-SY5Y, SK-N-AS) (Figure 1). The cell viabilities of all cell lines tested were greatly reduced with increasing concentrations of CFZ after being treated for 72 h (Figure 1A). The IC50s of CFZ in all six cell lines were calculated, ranged from 3.31 nM to 48.64 nM (Figure 1A). Our results indicate that CFZ inhibits cell growth in a dose-dependent manner in NB cell lines. In addition, the cytotoxic effect of CFZ in NB cells was further confirmed by cell morphology change after the treatment (Figure 1B).

### CFZ inhibits anchorage-independent growth of NB cells

**Figure 2:**
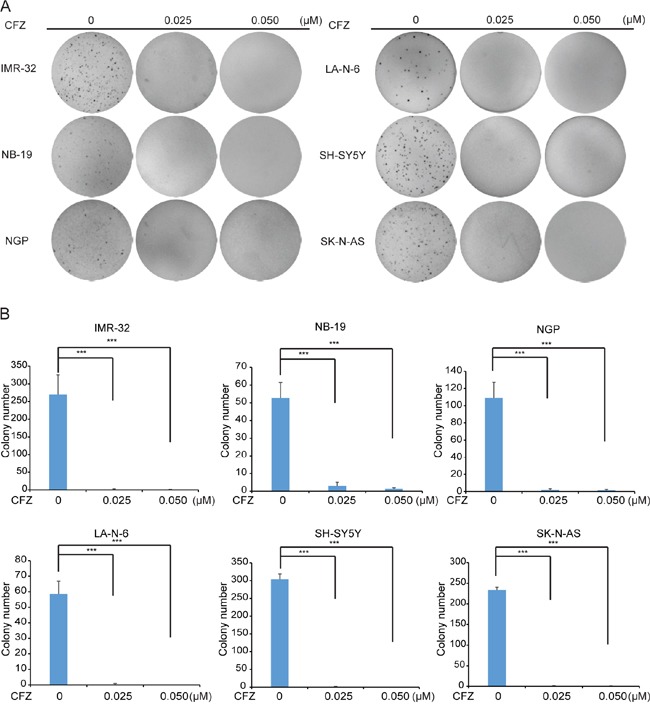
CFZ suppresses anchorage-independent growth of NB cells **A**. A panel of six NB cell lines were seeded in six-well plates with indicated concentrations of CFZ and agar, and grown for 2 to 3 weeks. Cells were stained with crystal violet for 4 h, and images were obtained. **B.** Colonies were counted and colony numbers were represented as mean ± SD with *P*<0.001 (***) (*ANOVA*, Dunnett) as indicated.

The anchorage-independent growth ability in soft agar is one of the characteristics of cancer cells. To evaluate whether CFZ could inhibit the anchorage-independent growth of NB cells, soft agar assays were performed. In all six cell lines, including IMR-32, NB-19, NGP, LA-N-6, SH-SY5Y, and SK-N-AS, a significant decrease in ability to form colonies were observed after CFZ treatment (0.025 μM, 0.05 μM), compared with vehicle-treated control (Figure 2A). The quantitative analysis also revealed the decreased colony numbers in CFZ treated cell lines (Figure 2B). Our results indicate that CFZ greatly attenuate anchorage-independent NB cell growth (Figure 2A and 2B).

### CFZ induces apoptosis in NB cells

**Figure 3:**
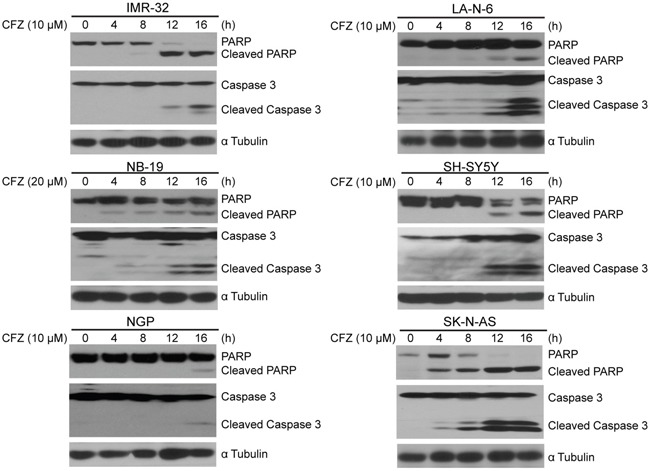
CFZ induces apoptosis of NB cells A panel of six cell lines were treated with 10 or 20 μM of CFZ for 0 h, 4 h, 8 h, 12 h or 16 h, subjected to SDS-PAGE, and then immunoblotted with anti-PARP, anti-Caspase 3, and anti-α Tubulin antibodies.

CFZ has been reported to induce apoptosis in a variety of tumor types, such as lung cancer and chronic lymphocytic leukemia [41]. Consistent with these reports, we found that CFZ treatment caused obvious Caspase 3 and PARP cleavage in NB cells tested (Figure 3), indicating CFZ induces apoptosis in NB cells. However, we noticed that two N-myc amplified cell lines NGP and NB-19 were more resistant to CFZ treatment compared to other NB cell lines tested (Figure 3).

### CFZ enhances the cytotoxic effect of Dox on NB cells

**Figure 4:**
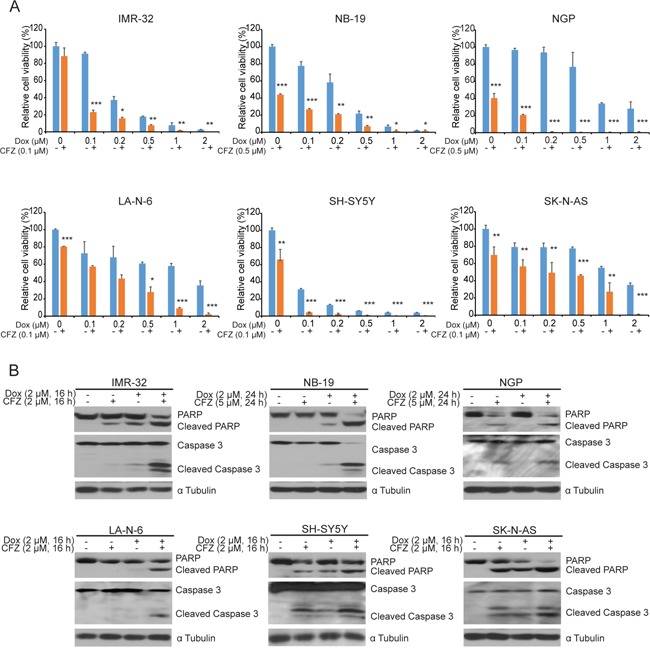
CFZ enhances the cytotoxic effect of Dox on NB cells **A.** Six NB cell lines were treated with increasing concentrations of Dox or combined with CFZ (0.1 μM or 0.5 μM) for 48 h. Cell viability was assessed by CCK-8 assay with *P*<0.05 (*), *P*<0.01 (**), or *P*<0.001 (***) (Student's t-test, two-tailed) as indicated. **B.** Six cell lines were treated with either Dox (2 μM) alone, CFZ (2 μM or 5 μM) alone, or a combination of both for a fixed 16 h. Then, they were subjected to SDS-PAGE and immunoblotted for anti-PARP, and anti-Caspase 3 antibodies. The anti-α Tubulin antibody was used as a loading control for whole cell extracts in all samples.

Since monotherapies may result in the development of cancer chemo-resistance, the better strategies in attacking them might be combination therapy. We then evaluated the combination effects of CFZ and the conventional anti-NB chemotherapy drug Dox on a panel of six NB cell lines: IMR-32, NB-19, NGP, LA-N-6, SH-SY5Y, and SK-N-AS. We observed enhanced cytotoxic effects on NB cells when cells were treated with CFZ in combination with Dox (Figure 4A). The combination treatment of CFZ and Dox also caused increased Caspase 3 and PARP cleavages compared to Dox or CFZ treatment alone (Figure 4B). These results demonstrate that CFZ greatly enhanced Dox-induced apoptosis in all NB cell lines.

### CFZ inhibits Dox-induced IκBα degradation and enhances Dox-induced p38 and JNK phosphorylation in NB cells

**Figure 5:**
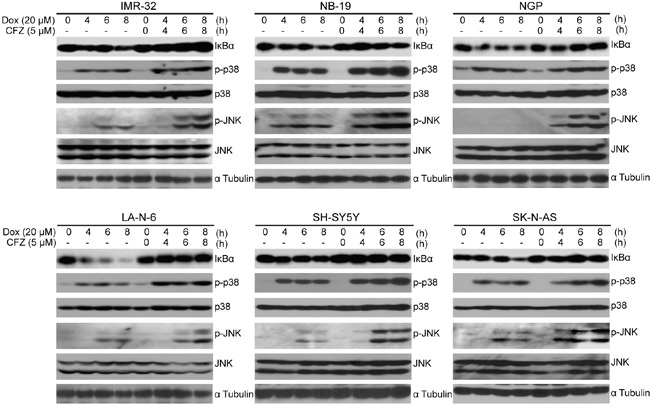
CFZ inhibits Dox-induced IκBα degradation and enhances Dox-induced p38 and JNK phosphorylation in NB cells A panel of six cell lines were treated with 20 μM of Dox or the combination of 10 μM CFZ and 20 μM Dox for 0 h, 4 h, 6 h or 8 h points, subjected to SDS-PAGE, and then immunoblotted with anti-IκBα, anti-p-p38, anti-p38, anti-p-JNK, anti-JNK and anti-α tubulin antibodies.

The NF-κB transcription factor induces the expression of a wide variety of genes important for cellular proliferation and survival, as well as inflammation and angiogenesis in NB [17, 21, 42]. Proteasome inhibition is a well-established approach which interferes with the NF-κB pathway by stabilizing IκBα. Therefore, in order to determine the mechanism of CFZ's cytotoxic effect on NB, we utilized immunoblotting analysis to test the expression of IκBα with single Dox (a strong NF-κB inducer) or Dox and CFZ combination treatment in a panel of six NB cell lines. Our data indicate that CFZ inhibited the Dox-induced IκBα degradation in all six cell lines tested (Figure 5). In addition, CFZ enhanced Dox-induced p38 and JNK phosphorylation (Figure 5). Together, our results indicate that CFZ may promote Dox-induced apoptosis by both enhancing p38 and JNK activation, and inhibiting NF-κB activation through blocking IκBα degradation.

### CFZ induces apoptosis *in vivo*

**Figure 6:**
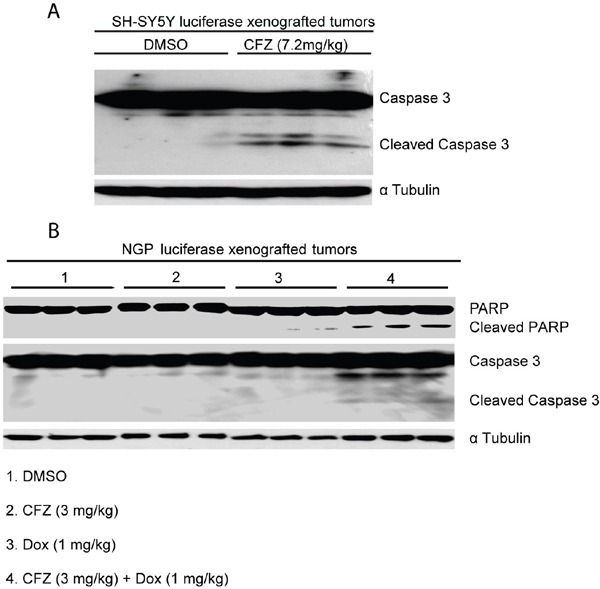
CFZ induces apoptosis in an orthotopic xenograft mouse model of NB **A.** The nude mice bearing SH-SY5Y NB cells were treated with DMSO or 7.2 mg/kg of CFZ (intraperitoneal injection) daily for 3 days. Then, the tumors were harvested and subjected to SDS-PAGE and immunoblotted for detecting Caspase 3 cleavage. The anti-α Tubulin antibodies was used as a loading control for whole cell extracts. **B.** The nude mice bearing NGP cells were treated with DMSO, 3 mg/kg of CFZ, 1 mg/kg Dox, and the combination of CFZ (3 mg/kg) and Dox (1 mg/kg) daily for 3 days, respectively. Then, the tumors were harvested and subjected to SDS-PAGE and immunoblotted for detecting PARP and Caspase 3 cleavages. The anti-α Tubulin antibodies was used as a loading control for whole cell extracts.

An orthotopic xenograft NB mouse model was utilized to test whether CFZ could induce apoptosis *in vivo*. Luciferase-transduced SH-SY5Y cells were surgically injected into the left renal capsule of nude mice. Five weeks after injection, tumor formation were detected by bioluminescent imaging. Mice were then randomly divided into two groups and treated with either dimethylsulfoxide (DMSO) (carrier control) or CFZ (intraperitoneally injected 7.2 mg/kg daily for 3 days). Then, tumors from the mice treated with CFZ and control were harvested and analyzed for apoptotic effectors by immunoblotting analysis. Consistent with the *in vitro* data, CFZ treatment caused Caspase 3 cleavage in tumor cells from CFZ treatment group *in vivo* (Figure 6A). In addition, owing that some N-myc amplified cell lines are more resistant to CFZ treatment compared to N-myc non-amplified cells, we tested whether CFZ could enhance the cytotoxic effects of Dox in N-myc amplified tumors *in vivo*. Using an orthotopic xenograft mouse model, we found that low dose of Dox (1 mg/kg) or CFZ (3 mg/kg) treatment did not cause detectable PARP and Caspase 3 cleavages whereas combination treatment with both CFZ and Dox caused obvious PARP and Caspase 3 cleavages (Figure 6B). These results suggest that CFZ could augment cytotoxic effects of Dox and enhance Dox-induced apoptosis *in vivo*.

## DISCUSSION

Proteasome in the ubiquitin-proteasome system mediates the cellular polyubiquitination of substrate proteins and proteolytic degradation. The inhibition of proteasome promotes the accumulation of cellular proteins which results in the vulnerability of cancer cells [43]. Therefore, the proteasome inhibitor presents itself as a promising anticancer agent. According to prior studies, proteasome inhibitors, such as Bortezomib, Ixazomib [44, 45], and Carfilzomib [26] [46, 47] have demonstrated therapeutic efficacy in a variety of cancer types. CFZ, which is approved by the FDA for treating the relapsed and refractory multiple myeloma, proved to be tolerable with high efficacy and safety in solid tumors, including lung cancer, head and neck squamous cell carcinoma, and glioblastoma [46] [48, 49]. Although the evaluation of CFZ and its mechanism on NB remain unknown, we hypothesize that CFZ might be an efficient and applicable anti-NB armamentarium.

Consistent with our hypothesis, we found that CFZ caused diminished cell proliferation and increased cell death in a panel of six NB cell lines, including LA-N-6, a well-known chemoresistant cell line. IC50 values for the tested cell lines treated with CFZ were all in the low nanomolar range (3.31 nM to 48.64 nM). This is similar to the reported CFZ IC50 values (0.2 nM to 99.4 nM) in other solid tumor cell lines [46, 47]. In addition, the soft agar assay revealed that CFZ strongly inhibits an anchorage-independent growth of NB cell lines. These results suggest that CFZ alone inhibits NB cell proliferation. After substantiating CFZ's inhibitory effects on NB cell proliferation, we also found that CFZ induced apoptosis in NB in all six cell lines tested.

Another critical issue frequently raised in the clinical setting is the drug resistance of monotherapies [50]. To overcome the potential chemoresistance built by single drug treatment, the combination of different chemotherapies may be a better treatment option for patients. Dox has been widely used as an anti-cancer agent for many tumor types, while Dox is reported to associate with multiple damaging side effects, especially injury to the heart [16]. Thus, finding ways to enhance the intended anti-cancer effects of Dox is vitally important. We evaluated the combinatory effects of CFZ and traditional drug Dox on NB. Our results indicate that in comparison to Dox alone, the combination of lower dose of CFZ and Dox achieved greater inhibitory effect on NB cell proliferation and enhanced PARP and Caspase 3 cleavages in NB cells, suggesting that CFZ is able to efficiently enhance the Dox-induced apoptosis in tumor cells and combination of CFZ and Dox may allow to use lower dose of Dox to achieve a better treatment outcome for NB patients with less Dox-caused side effect.

NF-κB activity has been implicated in chemoresistance of NB [18, 51], and CFZ is reported to interfere with the NF-κB activation in other cancer types. Since Dox serves as a strong NF-κB inducer and has been shown to activate NF-κB signal pathway in cancer cells [16], we tested whether CFZ could inhibit Dox-induced NF-κB activation in NB cells. Our results indicate that CFZ could inhibit Dox-induced NF-κB activation by stabilizing IκBα in NB cells. In addition, CFZ enhanced Dox-induced p38 and JNK phosphorylation. It is reported that p38 and JNK may mediate apoptotic pathway [52]. We reason that CFZ might enhance proteasome inhibition-induced cytotoxic stimuli and further counterbalance cellular homeostasis, which in turn stimulates higher phosphorylation and activation of p38 and JNK pathway. On the other hand, the CFZ-mediated inhibition of NF-κB activation might inhibit cell survival and enhance the efficacy of chemotherapy.

Moreover, CFZ showed anti-tumor efficacy in an orthotopic xenograft NB mouse model, since a relatively low concentration of CFZ (7.2 mg/kg) could induce apoptosis in N-myc non-amplified NB cell line SH-SY5Y *in vivo*. Some N-myc amplified cell lines are more resistant to CFZ treatment. In this case, it is likely that combination of CFZ and Dox could be a better strategy to treat N-myc amplified NB *in vivo*.

In summary, our results demonstrate that CFZ showed cytotoxic effects on NB by inducing apoptosis both *in vitro* and *in vivo*. Moreover, CFZ augmented Dox-induced cytotoxicity by enhancing Dox-induced p38 and JNK phosphorylation in a subset of NB cells. CFZ also overcome the chemoresistance of NB cells by stabilizing the protein level of IκBα and decreased Dox-induced NF-κB activity. The evaluation of proteasome inhibitor CFZ in NB sheds light on CFZ as a potential anti-NB armamentarium in combination with current chemotherapy.

## MATERIALS AND METHODS

### Antibodies and reagents

Anti-PARP (9532), anti-Caspase 3 (9662), anti-IκBα (9242), anti-phospho-p38 (9211), anti-p38 (9212), anti-phospho-JNK (9251), anti-JNK (9252), anti-mouse (7076) and anti-rabbit (7074) antibodies were from Cell Signaling Technology (Danvers, MA, USA); and anti-α Tubulin (10D8) (sc-53646) was from Santa Cruz Biotechnology (Dallas, TX, USA). CFZ was purchased from LC Laboratories (Woburn, MA, USA). Doxorubicin (D1515) was from Sigma-Aldrich Corp (St. Louis, MO, USA).

### Cell lines and cell culture

The human NB cell lines (IMR-32, NB-19, NGP, LA-N-6, SH-SY5Y and SK-N-AS) were cultured in RPMI 1640 medium (Lonza, Walkersville, MD, USA), supplemented with 20% or 10% (v/v) heat-inactivated Fetal Bovine Serum (FBS) (SAFC Biosciences, Lenexa, KS, USA), 100 units/mL penicillin, and 100 μg/mL streptomycin. All cells were maintained in a humidified incubator at a constant temperature of 37°C & 5% CO_2_. SH-SY5Y cell line with luciferase expression was generated with transduction of lentiviral luciferase virus containing a neo selectable marker, and then selected with G418800 μg/ml (Enzo Life Sciences, Farmingdale, NY, USA) for 10 days. All experiments were performed with indicated protocol [53].

### Cell viability assay

Cell viability was calculated using the Cell Counting Kit-8 (WST-8[2-(2-methoxy-4-nitrophenyl)-3-(4-nitrophenyl)-5-(2, 4-disulfophenyl)-2H-tetrazolium, monosodium salt]) (Dojindo Laboratories, Rockville, MA, USA). Cells were plated and grown in 96-well clear-bottom plates starting at 5 × 10^3^ cells/well. After 24 h of incubation, increasing concentrations of CFZ, Dox, or a combination of both were added to the wells and the cells were then incubated for another 48 h or 72 h. Then, a mixture of 10 μL of CCK-8 and 190 μL of medium with 10% or 20% FBS was added into each well respectively. After 1 h of incubation, the absorbance was measured at 450 nm using a microplate reader. Each experiment was performed in replicates of six and background reading of the medium was subtracted from each well to standardize the results.

### Cell imaging

A total of six NB cell lines (IMR-32, NB-19, NGP, LA-N-6, SH-SY5Y and SK-N-AS) were seeded in 96-well plates at appropriate concentrations [53]. After 72 h of treatment with indicated concentrations (0 μM, 0.005 μM, 0.05 μM) of CFZ, cell morphologies were observed and captured using an optical microscope.

### Colony formation assay

The soft agar assay for detecting colony formation abilities was performed as previously described [54]. A 5% (w/v) base agar layer was made by adding agar (214220, Difco Laboratories, Detroit, MI, USA) into distilled water and then the mixture were autoclaved for 50 min before cooling in a 56°C water bath. This solution was then mixed with medium and to a final concentration of 0.5%. To make the bottom agar layer, 2 mL of the 0.5% agar/medium solution were added to each well and cooled until semi-solid. The top agar layer was made of 0.3% agar 1.5 mL and each NB cell line was counted and added to the mixture at 1.5 × 10^4^ cells/well along with the indicated concentrations of CFZ. Cells were grown at 37°C for 2 to 3 weeks, then stained with 500 μL of 0.005% crystal violet (C3886, Sigma) for 4 h. Images were captured by the microscope, and colonies were counted [53]. Each assay was performed in triplicate.

### Immunoblotting assay

After each treatment, NB cells were washed twice with ice cold PBS. We collected cell pellets after centrifuging for 5 min at 6,000 rpm and then lysed on a rotator at 4°C for 30 min in cooled RIPA buffer (150 mM NaCl, 50 mM Tris-HCl at pH 7.4, 50 mM sodium fluoride, 1 mM EDTA, 1 mM dithiothreitol, 1 mM phenylmethylsulfonyl fluoride, 1 mM benzamidine, 0.1 mM sodium orthovanadate, 10 μg/mL leupeptin, 1% NP-40, 0.25% sodium deoxycholate, and phosphatase inhibitor cocktail 2 and 3 (p5726 and p0044, Sigma)). Then followed by centrifuging for 15 min at 13,000 rpm to collect supernatants as cell lysates. Protein concentration in cell lysates was measured using Bradford reagent (Bio-Rad Laboratories, Hercules, CA, USA), and samples were mixed with 4 × loading buffer, respectively, and heated at 100°C for 6 min. Lysates were separated by SDS-PAGE, transferred to polyvinylidence fluoride (PVDF) membranes (BioRad), blocked with 5% milk for 1 h at room temperature (25°C), and probed with appropriate dilutions of indicated primary antibodies overnight at 4°C. The membranes were then incubated with anti-mouse or rabbit IgG conjugated with horseradish peroxidase at room temperature for 1 h. The ECL-Plus Western detection system (GE Health Care, Buckinghamshire, UK) was then used for chemiluminescent visualization. The anti-α tubulin antibodies were used as a loading control for whole cell extracts in all samples.

### Antitumor efficacy in an orthotopic xenograft NB mouse model

Four to six-week-old female athymic NCR nude mice were purchased from Taconic (Hudson, NY, USA) and maintained under barrier conditions (pathogen-free conditions provided by plastic cages with sealed air filters). The preclinical mouse model of NB was established using orthotopic (intrarenal) implantation of the NB cells as described previously [53, 54]. Briefly, a transverse incision was created over the left flank of the nude mouse and 1.5 × 10^6^ human luciferase-transduced SH-SY5Y cells (N-myc non-amplified cells) and luciferase-transduced NGP cells (N-myc amplified cells) in 0.1 ml of PBS were surgically injected into the left renal capsule and toward the superior pole of the left kidney of the nude mice.

After engrafting for 5 to 6 weeks, mice bearing tumors with similar sizes (using bioluminescent imaging to monitor tumor growth) were randomly divided and treated with either DMSO or CFZ (7.2 mg/kg, intraperitoneal injection daily). For combination therapy, mice bearing tumors were treated with DMSO, CFZ (3 mg/kg), Dox (1 mg/kg) and CFZ (3 mg/kg) + Dox (1 mg/kg) every day. Three days later, the mice were sacrificed and the tumors were harvested and lysed for immunoblotting analysis. All mice were handled according to protocols approved by the Institutional Animal Care and Use Committee of the Baylor College of Medicine.

### Statistical Analysis

All values were presented as mean ± standard deviation (SD). A two-tailed Student's *t*-test and *ANOVA* were used to determine the statistical significance among drug treatment group and control group. Each assay was repeated at least twice, and representative results were presented. *P*<0.05 was considered to be statistically significant. The IC50 value was calculated by Graphpad Prism 5 software (La Jolla, CA, USA).

## Abbreviations

NB: neuroblastoma
CFZ: Carfilzomib
Dox: doxorubicin
DMSO: dimethyl sulfoxide
FDA: US Food and Drug Administration
NF-κB: the transcriptional factor nuclear factor kappa B
IκBα: the nuclear factor of kappa light polypeptide gene enhancer in B-cells inhibitor, alpha
PARP: poly ADP ribose polymerase.
